# Sound Radiation Analysis of Constrained Layer Damping Structures Based on Two-Level Optimization

**DOI:** 10.3390/ma12193053

**Published:** 2019-09-20

**Authors:** Dongdong Zhang, Yonghui Wu, Jingyue Chen, Shuwen Wang, Ling Zheng

**Affiliations:** 1 School of Mechanical Engineering, University of Shanghai for Science and Technology, Shanghai 200093, China; 182371466@st.usst.edu.cn (Y.W.); 183871584@st.usst.edu.cn (J.C.); wsw@usst.edu.cn (S.W.); 2 State Key Laboratory of Mechanical Transmission, Chongqing University, Chongqing 400044, China; zling@cqu.edu.cn

**Keywords:** constrained layer damping, two-level optimization, bi-directional evolutionary structural optimization, sound radiation power

## Abstract

Constrained layer damping (CLD) is an effective method for suppressing the vibration and sound radiation of lightweight structures. In this article, a two-level optimization approach is presented as a systematic methodology to design position layouts and thickness configurations of CLD materials for suppressing the sound power of vibrating structures. A two-level optimization model for the CLD structure is developed, considering sound radiation power as the objective function and different additional mass fractions as constraints. The proposed approach applies a modified bi-directional evolutionary structural optimization (BESO) method to obtain several optimal position layouts of CLD materials pasted on the base structure, and sound power sensitivity analysis is formulated based on sound radiation modes for the position optimization of CLD materials. Two strategies based on the distributions of average normalized elemental kinetic energy and strain energy of the base plate are proposed to divide optimal position layouts of CLD materials into several subareas, and a genetic algorithm (GA) is employed to optimally reconfigure the thicknesses of CLD materials in the subareas. Numerical examples are provided to illustrate the validity and efficiency of this approach. The sound radiation power radiated from the vibrating plate, which is treated with multiple position layouts and thickness reconfigurations of CLD materials, is emphatically discussed.

## 1. Introduction

Modern structures, involving thin plates and shell-type structures in vehicles, ships, and aerospace, are increasingly being made of lightweight materials due to the well-known social need for energy savings. Despite their superior structural characteristics, lightweight structures exhibit poor vibrational and acoustic isolation levels. The reduction of the vibration level and sound radiation in these lightweight structures can be achieved through damping treatments within acceptable limits. Constrained layer damping (CLD) treatment for the base structure has been regarded as an effective way to suppress structural vibration and acoustic radiation since CLD treatment was proposed by Kerwin [1]. The damping effect is caused by deformations of the soft viscoelastic layers, and enhanced by the constrained stiffer elastic layers. Owing to the real constraints of cost and weight in practical engineering structures, partial CLD treatment where only a portion of the base structure is covered with CLD is obviously more practical [2,3]. It is thus highly desirable to find the optimal configurations of CLD materials for the purpose of suppressing the vibration and sound radiation. This raises the problem of design optimization of the CLD materials attached to a base structure.

The optimum design of parameters for CLD structures, such as the thickness, densities, shear modulus, and coverage ratios, has been a major subject of research aiming to maximize modal damping ratios (MDRs), or minimize vibration energy and the vibration response. For example, Baz [4] optimized the performance of partially covered CLD beams by selecting the thickness of the viscoelastic core and length of CLD treatments, and Zheng [5] employed the genetic algorithm (GA) to optimize the locations and dimensions of CLD patches on a cylindrical shell for minimizing the vibration response. Sasikumar et al. [6] analyzed the effect of CLD parameters on the damping behavior for a beam-type structure based on the Taguchi method. The results demonstrated that the coverage of CLD has the most dominant effect on the total variation of the loss factor, followed by the constrained layer thickness and viscoelastic layer thickness. Recently, multi-objective optimization approaches [7,8] were also considered for maximizing modal loss factors for CLD-treated beams/plates.

More recent studies have applied topology optimization to find the optimal shape and positions of CLD materials on the base structure focusing on vibration suppression, while a specific type of CLD treatment with certain materials and dimensions along the thicknesses is considered. Kim [9] designed an interpolation scheme of the Rational Approximation of Material Properties (RAMP) for damping materials, and the Optimality Criteria (OC) method was adopted to obtain an optimal damping material layout for a shell structure to maximize the damping effect. The numerical results demonstrated that a higher modal loss factor can be achieved by the topology optimization method compared with that of the mode shape and the strain energy methods. Zheng, Xie and El-Sabbagh [10] developed a topology optimization model with maximizing modal damping ratios (MDRs) of elastic plates covered with CLD materials, and the Method of Moving Asymptote (MMA) was adopted to search for the best distribution of CLD treatments on plates. Kolk [11] proposed a method that applies multi-material, parametric level set-based topology optimization to simultaneously distribute structural and viscoelastic material to optimize damping characteristics. As an important methodology of topology optimization, the evolutionary structural optimization and bi-directional evolutionary structural optimization (ESO/BESO) method has since been extended for a wide range of topology optimization, such as stiffness optimization with multi-materials [12], the design of structural natural frequency [13], and the design of advanced materials [14]. Additionally, it is very natural to employ the ESO/BESO method in topology optimization problems for CLD structures. Liu and Xu [15] employed the ESO method to study the distribution optimization of CLD materials on different base structures with a consideration of maximizing the first modal loss factor. Fang [16] proposed a topology optimization model for CLD plates with the aim of minimizing the resonant response of the structure under specified broadband harmonic excitations, and ESO was employed to solve the problem. Furthermore, the ‘soft-kill’ BESO method was adopted to find the best distribution of CLD materials on the base plate [17]. Liu, Ruan and Huang [18] extended the bi-directional evolutionary structural optimization (BESO) method to design viscoelastic materials to optimize the damping and natural frequency of macrostructures by tailoring microstructures of viscoelastic materials.

From the works on CLD topology optimization focusing on vibration suppression, a summarized conclusion can be found that the CLD patches are optimally distributed on the area of a higher modal strain energy [19,20]. In the work on the topology optimization of CLD patches distributed on the base plate with respect to sound radiation [21], it is stated that a normal velocity distribution should be considered for reducing the sound power. Obviously, the sound radiation power of a structure depends not only on its structural damping, but also on its normal velocity profile. Hence, the optimal topology configurations of CLD treatments leading to the maximum modal damping ratios (MDRs) or the minimum vibration response, do not warrant a minimum sound radiation power of a flexible structure. Zhang [22] investigated the topology optimization of damping layers on the base structure under harmonic excitations for minimizing sound radiation, concluding that there are obvious differences between the optimal topologies obtained by minimizing the sound pressure and by minimizing the structural vibration.

As mentioned in the above works, both the optimum design of parameters and the topology optimization of CLD materials pasted on the base structure are useful for improving the performance of CLD structures, and the thicknesses of CLD materials are assumed to be pre-determined while topology optimization is carried out. Furthermore, it is difficult to optimally design thickness and position configurations of CLD materials for a complex structure in a topology optimization problem simultaneously. Hence, the author [23] proposed a two-level strategy to optimally design topology layouts and thicknesses of CLD materials simultaneously for reducing the sound power radiated from the vibrating structure. In the first level, the optimal position layouts of CLD patches are obtained by using the ESO method, which is limited by only allowing the removal of the CLD elements. In the second level, the obtained position layout is divided into two subareas based on the connectivity of CLD elements, and the thicknesses of CLD materials in the subareas are reconfigured. The numerical results demonstrated that an enhanced reduction of sound power can be achieved by comparing that induced by the optimal results of topology design.

In this paper, the work is extended based on [23], to present a systematic two-level optimization methodology to optimally design position layouts and thickness configurations of CLD materials for suppressing the sound power of vibrating structures. In first-level optimization, a modified bi-directional evolutionary structural optimization (BESO) method, which is characterized by a relaxed element removal/addition criterion, is presented to search for optimal position layouts of CLD materials pasted on the base structure. The two strategies that divide the optimal position layouts of CLD materials into subareas are proposed for reconfiguring thicknesses of CLD materials in second-level optimization. The layout of this paper is given as follows. Section 2 formulates the structural vibration and sound radiation power of CLD structures. Section 3 presents the two-level optimization model for CLD/plate considering two different additional mass fractions as constraints. Section 4 addresses the modified bi-directional evolutionary optimization (BESO) method for the first-level optimization and the two strategies to divide the position layouts of CLD materials. The corresponding two-level optimization procedure combining BESO and GA is summarized. Section 5 provides the numerical examples and discussions to demonstrate the effectiveness of the proposed approach. The conclusions are drawn in Section 6.

## 2. Structural Vibration and Sound Radiation of CLD/Plate

**Figure 1:**
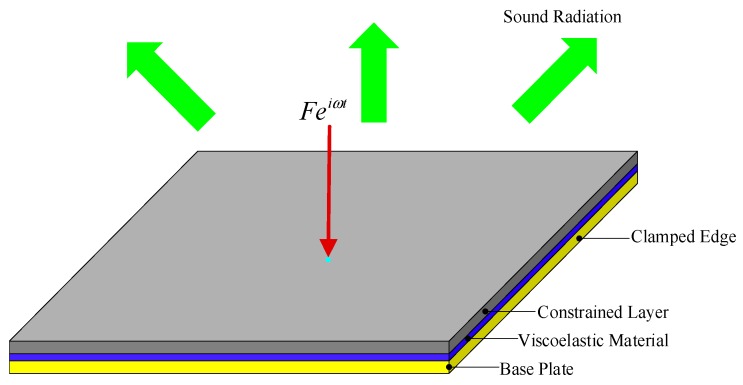
Illustration of sound radiation for a vibrating four-edge clamped plate treated with constrained layer damping (CLD) materials.

Figure 1 illustrates the sound radiation of a vibrating elastic plate treated with CLD materials. It is assumed that the plate is surrounded by an infinite baffled plate. The four edges of the base plate are clamped, and a harmonic force $f=Fe^{i\omega t}$ with a given natural frequency ω and amplitude F is applied at the center of the plate. The sound radiation power induced by the vibrating elastic plate to half-space is considered, while the structural–acoustic coupling effect is neglected. Firstly, the structural vibration of CLD/plate is analyzed by using the finite element method, and the sound radiation power is then derived using the boundary element method and sound radiation modes.

### 2.1. Structural Vibration Analysis of CLD/Plate

A complex constant model $G_{v}=G_{\infty }(1+\eta i)$ (where η is the material loss factor) is considered for the shear modulus of viscoelastic materials, and the dynamic equation of CLD/plate can then be written as

(1) $$M\ddot{y}(t)+\left(K+G_{v}K_{s}\right)y(t)=f\left(t\right)+LP_{s}(t)$$

in which **M** is the global mass matrix; **K** is the global stiffness matrix without considering the shear deformation of damping material; $K_{s}$ is the shear stiffness matrix of the damping material; y(t) and $\ddot{y}(t)$ denote the vectors of displacement and acceleration of the vibrating CLD/plate, respectively; **L** is the fluid-structural coupling matrix; and $P_{s}(t)$ represents the sound pressure induced by the acoustic medium acting on the plate surface. In this paper, the acoustic medium is air and we consider the coupling between the fluid and structure to be weak, so it can be neglected and the second term on the right-hand side of Equation (1) vanishes.

In Equation (1), the global mass matrix and stiffness matrix can be expressed as follows:

(2) $$M=\sum_{e=1}^{N}M_{e}^{b}+\sum_{e=1}^{N}M_{e}^{v}\left(h_{v}\right)+\sum_{e=1}^{N}M_{e}^{c}\left(h_{c}\right)$$

(3) $$K=\sum_{e=1}^{N}K_{e}^{b}+\sum_{e=1}^{N}K_{e}^{v}\left(h_{v}\right)+\sum_{e=1}^{N}K_{e}^{c}\left(h_{c}\right)$$

(4) $$K_{s}=\sum_{e=1}^{N}K_{se}^{v}\left(h_{v}\right)$$

in which $M_{e}^{b}$, $K_{e}^{b}$ represent the elemental mass matrix and stiffness matrix for the base plate, respectively; $M_{e}^{v}\left(h_{v}\right)$, $M_{e}^{c}\left(h_{c}\right)$ and $K_{e}^{v}\left(h_{v}\right)$, $K_{e}^{c}\left(h_{c}\right)$ denote the elemental mass matrix and stiffness matrix of the damping layer and constrained layer, respectively; and $K_{se}^{v}\left(h_{v}\right)$ is the elemental shear stiffness matrix for the damping layer. *N* is the total number of discretized elements for the CLD structure. To obtain the optimal thickness of CLD materials, the thickness variables, $h_{v}$ and $h_{c}$, of viscoelastic material (VEM) and constrained layer material (CLM), are separated from the corresponding matrices.

The steady-state response of the CLD/plate under forced excitation f(t) is expressed as

(5) $$y(t)={\left(-\omega^{2}M+\left(K+G_{v}K_{s}\right)\right)}^{-1}F$$

In order to calculate the sound power radiated from the vibrating structure, the normal vibration velocity vector $v_{nn}$ of the nodes needs to be obtained and can be formulated as

(6) $$v_{nn}=i\omega y_{nn}$$

where $y_{nn}$ is the normal displacement vector of the nodes, which can be obtained from Equation (5).

### 2.2. Sound Radiation Power Analysis of CLD/Plate

**Figure 2:**
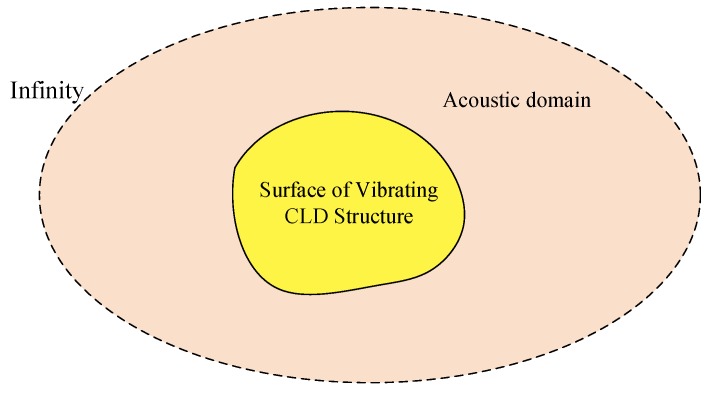
Exterior sound radiation problem.

Sound radiation of the CLD/plate structure in this paper could be considered as an exterior sound radiation problem, as schematically shown in Figure 2. The CLD/plate structure is embedded in an infinite sound field and discretized by the finite element method, and the acoustic domain is coupled with the plate structure, i.e., the plate surface is discretized using boundary elements. Sound power radiated from the vibrating plate structure, which represents the sound radiation capability of the vibration structure, is selected as the optimization objective in the following sections.

For the vibrating plate structure, the sound radiation power can be expressed using the sound pressure p(α) on the structure’s surface and the surface normal velocity $v_{n}(\alpha )$.

(7) W=12∬SRe[p(α)vn*(α)]dS(α)

Here, $v_{n}^{*}(\alpha )$ represents the complex conjugate of the surface normal velocity $v_{n}(\alpha )$.

While the plate structure and its surface are discretized, the sound power can be formulated using the sound radiation mode approach [24]:

(8) $$W(\omega )=v_{n}^{H}Rv_{n}$$

in which *H* is a complex conjugate, and $v_{n}$ represents the normal vibration velocity vector of the elements. It is reasonable that the normal velocity at any point of an element is assumed to be equal due to the small element area ***s****_e_*. From this point of view, the normal velocity at an element’s center, which can be easily obtained by interpolation of the normal velocity $v_{nn}$ of the nodes, is employed to represent the elemental normal velocity $v_{n}$. **R** is the surface radiation resistance matrix and can be written as

(9) $$R=\frac{\rho \omega^{2}s_{e}^{2}}{4\pi c}\left[\begin{array}{cccc} 1 & \frac{\sin(kr_{12})}{kr_{12}} & \cdots & \frac{\sin(kr_{1N})}{kr_{1N}}\\ \frac{\sin(kr_{21})}{kr_{21}} & 1 & \cdots & \cdots \\ \vdots & \cdots & \cdots & \cdots \\ \frac{\sin(kr_{N1})}{kr_{N1}} & \cdots & \cdots & 1 \end{array}\right]$$

where $r_{mn}$ is the distance between the center of element *m* and element *n*.

In the low frequency range, the sound radiation of the vibrating plate structure is mainly contributed by low-order sound radiation modes. Hence, the sound radiation power of the structure can be approximately written as the sum of several low-order sound radiation modes:

(10) $$W(\omega )={\left(Q^{T}v_{n}\right)}^{H}\Lambda Q^{T}v_{n}=q^{H}\Lambda q=\sum_{i=1}^{MP}\lambda_{i}{\left|q_{i}\right|}^{2}$$

in which **Q** and Λ are obtained from eigenvalue decomposition $R=Q\Lambda Q^{T}$; $Q=\left[\begin{array}{cccc} Q_{1} & Q_{2} & \cdots & Q_{N} \end{array}\right]$ is defined as the sound radiation mode matrix; $\Lambda =\mathrm{diag}\left[\begin{array}{cccc} \lambda_{1} & \lambda_{2} & \cdots & \lambda_{N} \end{array}\right]$ represents the radiation coefficients matrix of the corresponding radiation modes; $q=Q^{T}v_{n}$ is defined as the adjoint matrix of radiation modes; $q_{i}$ is an adjoint coefficient of the *i*th radiation mode $Q_{i}$; and *MP* is the number of radiation modes in a low frequency range.

## 3. Two-Level Optimization Model for CLD/Plate

It is known that the position layouts and thicknesses of CLD materials pasted on the base structure have important influences on the reduction of sound power. Hence, it is necessary to carry out coordinated optimization design for positions and thicknesses of CLD materials on the base structure. In this paper, a two-level progressive optimization model is proposed for searching for optimal configurations of positions and thicknesses for CLD materials, with the aim of minimizing the sound power of the vibrating structures with a given amount of CLD materials in a prescribed design domain, as schematically illustrated in Figure 1.

In the first level, position optimization for CLD materials on the base structure is naturally considered as a topology optimization problem, and is expressed as follows:

(11) $$\begin{array}{cc} find: & x=\left\{x_{1},\;x_{2},\;\cdots ,\;x_{Ne}\right\}\\ min: & W\left(x\right)\\ subject\;to: & \sum_{i=1}^{N_{1}}x_{i}\left(\rho_{c}h_{ci}+\rho_{v}h_{vi}\right)s_{e}/m\leq \delta_{1}\\ & x_{i}=\left\{0,\;1\right\},\;i=1,\;2,\;\cdots ,Ne;\;h=\left\{h_{vi},\;h_{ci}\right\}=constant \end{array}$$

where $x=\left\{x_{1},\;x_{2},\;\cdots ,\;x_{Ne}\right\}$ is a vector used to describe the existence states of CLD elements; $x_{i}=1$ indicates that the *i*th CLD element is considered as a solid element, which is pasted on the base plate, and on the contrary, $x_{i}=0$ means that the *i*th CLD element is a void element, which is deleted from the base plate; *W* is the sound radiation power induced by vibrating the CLD/plate; *N*_1_ is the total number of CLD elements; *m* is the total mass of CLD materials which are fully pasted on the base plate; $\delta_{1}$ is a given additional mass fraction which can be used as an initial value for the second-level optimization; $s_{e}$ is the surface area of each CLD element; and $h=\left\{h_{vi},\;h_{ci}\right\}$ denotes the thickness of viscoelastic damping material and constrained layer material for the *i*th element, and they are assigned as initial values in first-level optimization.

In second-level optimization, finding optimal configurations of thicknesses of VEM and CLM will be carried out. Specifically, the optimal CLD material layouts, which are obtained from the first-level stage and considered as the initial design, are divided into several subareas, and the thicknesses of VEM and CLM for the subareas are optimally reconfigured. The optimization problem is described as follows:

(12) $$\begin{array}{cc} find: & h=\left\{\begin{array}{cccccc} h_{v1} & h_{c1} & \cdots & \cdots & h_{vn} & h_{cn} \end{array}\right\}\\ min: & W\left(h\right)\\ subject\;to: & x=\left\{x_{1},\;x_{2},\;\cdots ,\;x_{Ne}\right\}=\mathrm{constant}\\ & \sum_{i=1}^{N_{2}}\left(\rho_{c}h_{ci}s_{e}+\rho_{v}h_{vi}s_{e}\right)/m\leq \delta_{2},\;h_{c.v}=\left\{h_{min},\;h_{max}\;\right\} \end{array}$$

where $h=\left\{\begin{array}{cccccc} h_{v1} & h_{c1} & \cdots & \cdots & h_{vn} & h_{cn} \end{array}\right\}$ is a vector of the thickness variables for the *n* subareas; *N*_2_ is the total number of CLD elements pasted on the optimal layouts obtained from the first-level optimization; and $\delta_{2}$ is the target additional mass fraction for the final configurations of CLD/plate. It is noted that the additional mass fraction $\delta_{1}$ assigned in first-level optimization can be larger or smaller than $\delta_{2}$ in second-level optimization.

## 4. Optimization Method and Procedure

**Figure 3:**
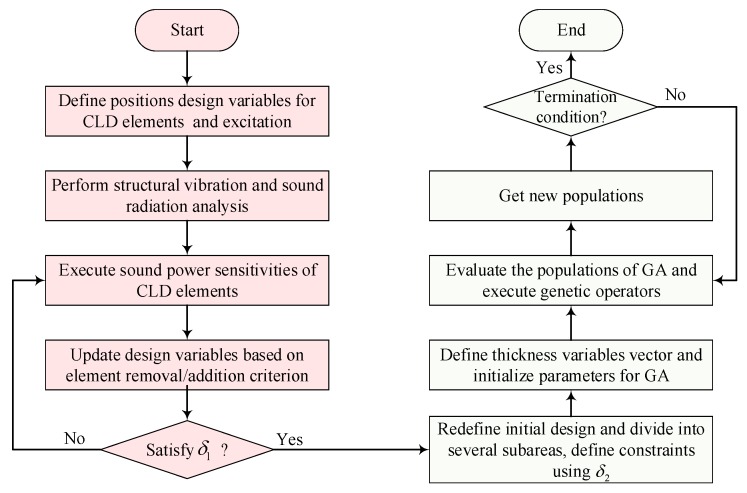
Flowchart of the proposed two-level optimization.

In first-level optimization, the convergent and mesh-independent BESO method [25] is applied and modified to obtain optimal topology configurations of CLD materials on the base structure. In second-level optimization, by dividing the optimal CLD layouts into several subareas based on the average normalized elemental kinetic energy and strain energy of the base plate, the optimal thickness of CLD materials for each subarea can be searched for using the genetic algorithm (GA). The two-level optimization procedure is implemented in MATLAB R2016b (Albuquerque, NM, USA, and the flowchart is given in Figure 3. The details are further explained in the following subsections.

### 4.1. First-Level Optimization

#### 4.1.1. Sound Power Sensitivity Analysis

Sensitivity analysis is necessary for providing the descent search direction of the defined problem in first-level optimization. Hence, according to Equation (10), the sound radiation power sensitivity with respect to the CLD element positions $x=\left\{\begin{array}{cccc} x_{1} & x_{2} & \cdots & x_{m} \end{array}\right\}$ can be defined as follows:

(13) $$\frac{\partial W}{\partial x_{m}}=\sum_{i=1}^{MP}\left(\frac{\partial \lambda_{i}}{\partial x_{m}}{\left|q_{i}\right|}^{2}+\lambda_{i}\frac{\partial q_{i}^{*}}{\partial x_{m}}q_{i}+\lambda_{i}q_{i}^{*}\frac{\partial q_{i}}{\partial x_{m}}\right)$$

in which $q_{i}^{*}$ is the complex conjugate of $q_{i}$. Substituting $q_{i}=Q_{i}^{T}v_{n}$ into Equation (13), and considering that the influence of design variable $x_{i}$ on $\lambda_{i}$ and $Q_{i}$ can be neglected in the low frequency range [26], Equation (13) can be rewritten as

(14) $$\frac{\partial W}{\partial x_{m}}=\sum_{i=1}^{MP}\left(\lambda_{i}{\left(Q_{i}^{T}\frac{\partial v_{n}}{\partial x_{m}}\right)}^{*}q_{i}+\lambda_{i}q_{i}^{*}Q_{i}^{T}\frac{\partial v_{n}}{\partial x_{m}}\right)$$

In Equation (14), the normal velocity sensitivity with respect to the design variable is generally difficult to obtain directly. In this paper, the following approximate method is adopted, which is derived from Equation (6). Hence, the element normal velocity sensitivity is formulated as follows:

(15) $$\frac{\partial v_{n}}{\partial x_{m}}=\frac{s_{T}\partial v_{nn}}{\partial x_{m}}=i\omega \frac{s_{T}\partial y_{nn}}{\partial x_{m}}$$

where $s_{T}$ is the shape function matrix which transforms the nodal normal velocity vector $v_{nn}$ to the elemental normal velocity vector $v_{n}$. According to Equations (15) and (14), the sound radiation power sensitivity with respect to the design variable is given as follows:

(16) $$\frac{\partial W}{\partial x_{m}}=i\omega \sum_{i=1}^{MP}\left(\lambda_{i}{\left(Q_{i}^{T}\frac{s_{T}\partial y_{nn}}{\partial x_{m}}\right)}^{*}q_{i}+\lambda_{i}q_{i}^{*}Q_{i}^{T}\frac{s_{T}\partial y_{nn}}{\partial x_{m}}\right)$$

The sensitivity of nodal normal displacement in Equation (16) is derived from Equation (5):

(17) $$\frac{\partial y_{nn}}{\partial x_{m}}=-i\omega {\left(\left(K+G_{v}K_{v}\right)-\omega^{2}M\right)}^{-1}\left(\frac{\partial \left(K+G_{v}K_{v}\right)}{\partial x_{m}}-\omega^{2}\frac{\partial M}{\partial x_{m}}\right)y_{nn}$$

in which the derivatives of the mass matrix and stiffness matrix to design variables can be approximately calculated using the difference method.

#### 4.1.2. Optimization Flowchart of Improved BESO

The evolutionary structural optimization (ESO) method proposed by Xie and Steven [27] in the early 1990s, which is characterized by its simplicity for topology optimization, has since been extended for a wide range of topology optimization [28]. The traditional ESO procedure only allows the material elements to be deleted from the finite element model of the structure monotonically, making it impossible to replenish improperly removed material elements. To deal with this problem, Querin and Xie [29] proposed bi-directional evolutionary structural optimization, which is capable of adding and deleting material elements simultaneously. The latest version of the BESO method, i.e., the convergent and mesh-independent BESO method [25], has been applied for various structural design problems, such as the design of structural natural frequency [13,30] and the design of nonlinear structures [31].

The BESO method in reference [25] is also employed in first-level optimization. At each iteration, the procedure employed to calculate the sound power sensitivity, usually in terms of a complex equation, is computationally expensive. Hence, reducing the number of iterations as much as possible subjecting to the given constraints will cut down the computational effort for the whole optimization procedure. Here, a relaxed element removal/addition criterion is proposed to modify the BESO, leading to a reduction in the number of iterations. In addition, a smaller additional mass fraction, $\delta_{1}$ ($\delta_{1}$ < $\delta_{2}$), is employed to obtain several optimal distributions of CLD materials, which will be considered as the initial design for thickness reconfigurations. A general flowchart for the first-level optimization problem is explained as follows:

**Step 1.** The plate fully treated with CLD materials is defined as the initial design, and a composite four-node CLD element is used to discretize the initial design for the given boundary conditions. Design variables (0 or 1) for the elements of the initial design are assigned;
**Step 2.** The stiffness and mass matrices for CLD/plate using the current design variables are determined, and the dynamic equation is solved to obtain the resonant frequency. Then, a harmonic force with a specified resonant frequency is defined to excite the plate at a specified point;
**Step 3.** Finite element analysis is performed to obtain the node displacement, and the normal velocity vector is calculated using Equation (5). Then, the sound power for the current design can be obtained;
**Step 4.** Elemental sensitivities of the sound power with respect to the design variables are analyzed using Equation (16). Based on the method in reference [25], the elemental sensitivities are transformed to nodal sensitivities using Equation (18), and are then converted into elemental sensitivities using Equation (19), through which the sensitivity numbers for void elements are extrapolated. Following this, the elemental sensitivity number is further smoothed by averaging that of the current and previous iteration.
  (18) $$s_{j}^{n}=\sum_{i=1}^{M}V_{i}s_{i}^{e}/\sum_{i=1}^{M}V_{i}$$
  (19) $$s_{i}^{ne}=\sum_{i=1}^{M_{r}}\left(r_{\min}-r_{ij}\right)s_{j}^{n}/\sum_{i=1}^{M_{r}}\left(r_{\min}-r_{ij}\right)$$
  In Equations (18) and (19), $s_{j}^{n}$ is the sensitivity of the *j*th node; $s_{i}^{e}$ and $s_{i}^{ne}$ denote the *i*th element sensitivity before and after being transformed, respectively; *V_i_* is the volume of the *i*th element; *M* is the total element number connected to the *j*th node; *M_r_* is the total nodes number in the circular domain with the radius *r*_min_; and $r_{ij}$ is the distance between the central element *i* and *j*th node;
**Step 5.** The deleting elements and adding elements are determined using the element removal/addition criterion detailed in Section 4.1.3. For deleting elements (solid elements), the property value is switched from 1 to 0, and for adding elements (void elements), it is switched from 0 to 1;
**Step 6.** Steps 2–5 are repeated until the given additional mass fraction ratio $\delta_{1}$ is satisfied.

#### 4.1.3. The Element Removal/Addition Criterion

For BESO in reference [25], an element removal/addition criterion is developed by using a threshold sensitivity, which is defined according to a fixed volume/mass constraint at each iteration. Here, the element removal/addition criterion in original BESO is improved, in which a variable threshold sensitivity is defined to relax the element removal/addition at each iteration. This will reduce the number of iterations required for the optimization procedure. The details of this procedure are as follows:

**Step 1.** The target mass/volume for the next iteration is given first by using the number of void elements:
  (20) $$N_{k+1}=N_{k}+N_{d}$$
  where $N_{k+1}$ and $N_{k}$ are the number of void elements at iteration *k* + 1 and *k*, respectively, and $N_{d}$ is a variable number, denoting the number of elements which will be switched to void elements;
**Step 2.** An initial threshold sensitivity $S_{ak}$ for the *k*th iteration is determined by averaging the non-zero sensitivities of all elements (solid and void);
**Step 3.** The solid elements are removed and the void elements are recovered by the following steps:
  (1) For the *i*th solid element, if the sensitivity $S_{si}$ satisfies Equation (21), then the *i*th solid element is removed to be a void element. In Equation (21), $\alpha_{s}$ is an adjustable positive number used to ensure that the number of removed solid elements $N_{s}\leq N_{smax}$, and $N_{smax}$ is introduced to ensure that not too many solid elements are removed at each iteration;
    (21) $$S_{si}\geq \left(1\pm \alpha_{s}\right)S_{ak}$$
  (2) For the *i*th void element, if the sensitivity $S_{vi}$ satisfies Equation (22), then the *i*th void element is switched to be a solid element. In Equation (22), $\alpha_{v}$ is also a positive number used to ensure that the number of removed solid elements $N_{v}\leq N_{vmax}$, and $N_{vmax}$ is introduced to ensure that not too many void elements are recovered at each iteration.
    (22) $$S_{vi}\leq \left(1\mp \alpha_{v}\right)S_{ak}$$
    It is noted that the initial threshold sensitivity $S_{ak}$ for CLD elements may be greater or less than zero, hence the plus sign is used while $S_{ak}$ is greater than zero; otherwise, a minus sign is used in Equation (21), which is contrary to Equation (22). In addition, $N_{vmax}<N_{smax}$ is required to ensure $N_{d}=N_{s}-N_{v}$ elements are removed at each iteration;
  (3) If $N_{s}>N_{v}$ cannot be reached in steps (1) and (2), the first $N_{smax}$ solid elements can be removed according to the sensitivities in a descending order and the first $N_{vmax}$ void elements can be recovered according to the sensitivities in an ascending order. Then, $N_{d}=N_{smax}-N_{v\max}$ elements become void elements.

### 4.2. Second-Level Optimization

The CLD material layout obtained in first-level optimization, which will be divided into *n* reasonable subareas, is considered as the initial design for the second-level optimization. The corresponding additional mass fraction is denoted by $\delta_{i}$. Hence, the issues relating to how to divide the initial design domain (optimal position layouts of CLD materials) into reasonable subareas in the second level should be emphasized. In many references [19,20] focusing on vibration suppression, it can be seen that the CLD patches are optimally distributed on the area of a higher modal strain energy, and a normal velocity distribution should be addressed for reducing the sound power [32,33]. Activated by the points, the optimal CLD material layouts can be divided into *n* reasonable subareas based on the distributions of elemental strain energy and elemental kinetic energy, and a design variable vector consisting of the thicknesses for the *n* subareas can be defined as follows:

(23) $$h=\left\{\begin{array}{cccccc} h_{v1} & h_{c1} & \cdots & \cdots & h_{vn} & h_{cn} \end{array}\right\}$$

Owing to the ergodicity of evolution operations and robustness for a global search, a genetic algorithm (GA) in reference [19] is applied to search for the optimum thicknesses of CLD materials. The combination of a normalized constraint violations value and a penalty function is used to guarantee that the additional mass fraction ratio changes from the initial value $\delta_{i}$ to the target value $\delta_{2}$. In the evolution process, if the minimum sound power is no longer changed for the ten consecutive generations, the evolutionary process is terminated in advance; otherwise, while the specific maximum number of generations is achieved, the evolutionary process is terminated.

## 5. Numerical Example and Analysis

A rectangle CLD/plate in an infinite baffle plate in which four edges of the base plate are clamped is considered, as shown in Figure 1. The proposed two-level optimization approach in Section 4 is adopted to obtain optimal configurations of CLD materials attached to the plate, and the discussions of optimization results are emphasized. The base plate (aluminum) dimensions are 400 × 300 × 0.8 mm, and it has a Young’s modulus of E_b_ = 7.0 × 10^10^ N/m^2^, Poisson’s ratio of $\mu_{b}$ = 0.3, and mass density of $\rho_{b}$ = 2800 kg⁄m^3^. The constrained layer material (CLM) has a Young’s modulus of E_c_ = 7.0 × 10^10^ N/m^2^, Poisson’s ratio of $\mu_{c}$ = 0.3, and mass density of $\rho_{c}$ = 2800 kg⁄m^3^. The viscoelastic material (VEM) has a shear modulus of G_v_ = 1.2 × 10^7^ N/m^2^, Poisson’s ratio of $\mu_{v}$ = 0.5, and mass density of $\rho_{v}$ = 1200 kg⁄m^3^. The thicknesses of CLM and VEM are assigned by 0.1 mm, defined as the initial value in the first-level optimization.

The CLD/plate is discretized into 32 × 24 finite element meshes by using a four-node quadrilateral composite element [23] with seven degree of freedoms (DOFs) per node, and the uniform-sized boundary elements are used at the upper surface of the CLD/plate. The fully treated CLD/plate is selected as the initial design, and the target additional mass fraction is set to be $\delta_{2}$ = 0.5 in the global optimization procedure. In the first-level optimization process, the additional mass fraction is set to be $\delta_{1}$ = 0.2, to try to obtain several optimal CLD position layouts; the target additional mass fraction is achieved by redistributing the thicknesses of CLM and VEM for several optimal CLD position layouts. The two-level optimization procedure is implemented in MATLAB, and the results and discussions are illustrated in the following subsections.

### 5.1. Results and Discussion for First-Level Optimization

The modified BESO in Section 4.1 is employed to find the optimal position layouts of CLD materials. The maximum number of removed solid CLD elements $N_{smax}$ = 32, and the maximum number of recovered void elements $N_{vmax}$ = 4. The filter radius in Equation (19) $r_{\min}$ = 3 × *l*_e_, in which *l*_e_ denotes the element dimension.

**Figure 4:**
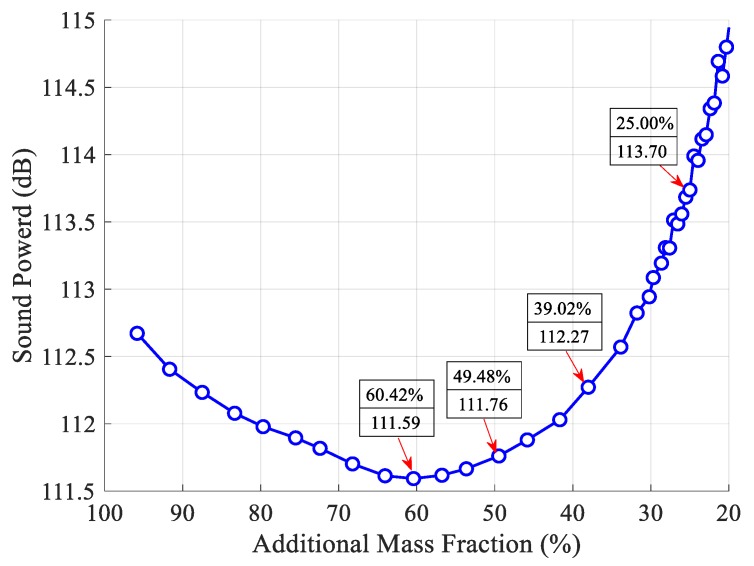
Iteration histories of sound power with additional mass fraction ratio.

**Figure 5:**
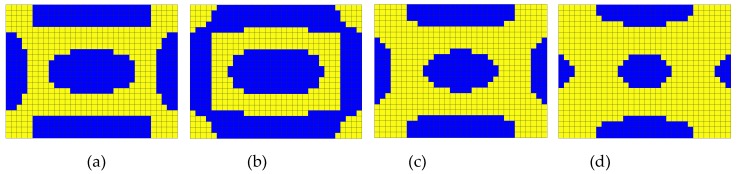
Optimal position layouts for CLD materials with different additional mass fraction ratios (‘blue’ denotes CLD materials, ‘yellow’ denotes base plate). (**a**) Additional mass fraction (AMF) = 60.42%; (**b**) AMF = 49.48%; (**c**) AMF = 39.02%; (**d**) AMF = 25%.

The iteration history of sound power with additional mass fraction ratios is presented in Figure 4. It is very clear that the additional mass fraction ratios are not evenly spaced, which implies that removing solid CLD elements during an earlier stage of the optimization process plays a key role in suppressing the sound radiation power, and in contrast, recovering void elements during a later stage of optimization for improving the reduction of sound radiation power is very useful. It can also be inferred that the number of iterations has been reduced compared to that induced by a fixed threshold sensitivity, which implies that the computational effort required for global optimization is decreased. Furthermore, an optimal additional mass fraction (AMF), 60.42%, which corresponds to an optimal position layout of CLD materials shown in Figure 5a, can be found to maximize the sound power reduction in Figure 4. Then, for comparison, the other three position layouts for CLD materials are also selected as the initial design in the second-level optimization, shown in Figure 5b–d. Therefore, it is very natural that the additional mass fractions of the optimal layouts in Figure 5a–d are defined as the initial mass fractions in second-level optimization.

Besides, the modal damping ratios (MDRs) corresponding to the optimal CLD layouts in Figure 5 are also monitored. For comparison, Table 1 lists the sound power and MDRs for the optimal CLD layouts in Figure 5 and the fully treated CLD/plate. One can observe from Table 1 that compared with those of the fully treated CLD/plate, both the sound power and vibration level of the plate can be efficiently suppressed with optimal partially-covered CLD treatments.

### 5.2. Results and Discussion for Second-Level Optimization

**Figure 6:**
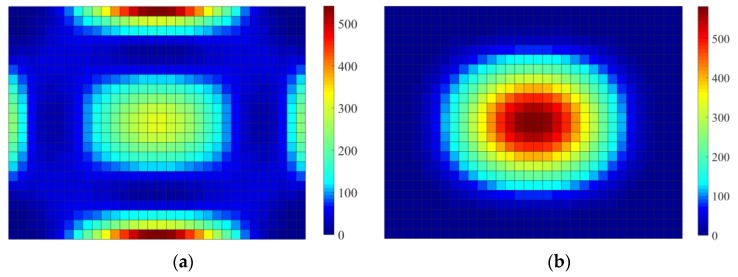
Elemental strain energy and kinetic energy distributions of the base plate. (**a**) Distributions of elemental strain energy; (**b**) distributions of elemental kinetic energy.

Figure 6 shows the distributions of elemental strain energy and kinetic energy of the base plate. Comparing Figure 5 and Figure 6, the same conclusion described in Section 4.2 can be obtained, i.e., the distributions of strain energy and element kinetic energy should be addressed for reducing the sound radiated power of vibrating structures. Hence, when activated by the point, the initial design domain is divided into two or three subareas based on the distributions of elemental kinetic energy and strain energy of the base plate. Furthermore, the thicknesses of CLM and VEM pasted on the subareas are optimally reconfigured. In the following subsections, the thickness range of CLM and VEM is set to be from 0.01 to 0.2 mm.

#### 5.2.1. Thickness Optimization for Case 1 and Case 2

**Figure 7:**
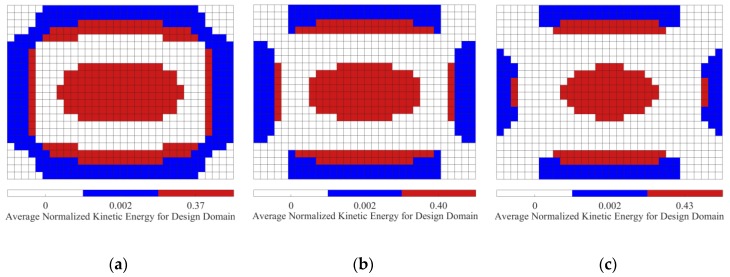
Diagram of the two subareas divided based on distributions of average normalized element kinetic energy (‘red’—subarea $S_{1}^{ke}$ with a higher element kinetic energy; ‘blue’—subarea $S_{2}^{ke}$ with a lower element kinetic energy). (**a**) AMF = 60.42%; (**b**) AMF = 49.48%; (**c**) AMF = 39.02%.

**Figure 8:**
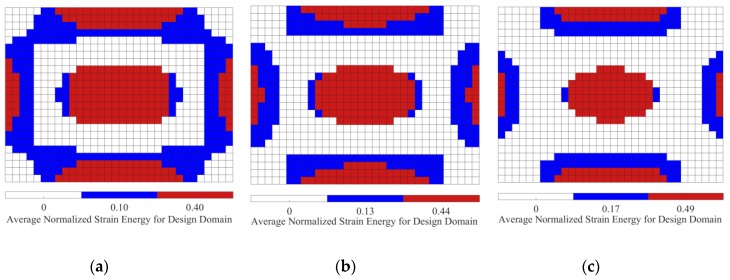
Diagram of the two subareas divided based on distributions of average normalized element strain energy (‘red’—subarea $S_{1}^{se}$ with a higher element strain energy; ‘blue’—subarea $S_{2}^{se}$ with a lower element strain energy). (**a**) AMF = 60.42%; (**b**) AMF = 49.48%; (**c**) AMF = 39.02%.

We now consider the optimal position layouts of CLD materials, shown in Figure 5a–c, as the initial design domains for second-level optimization. They are divided into two equal subareas based on the distributions of average normalized elemental kinetic energy and strain energy of the base plate, illustrated in Figure 7 and Figure 8, in which the ‘red’ color denotes the subarea $S_{1}^{ke}$ and $S_{1}^{se}$ with a higher elemental kinetic energy and strain energy, and the ‘blue’ color means the subarea $S_{2}^{ke}$ and $S_{2}^{se}$ with a lower elemental kinetic energy and strain energy. For convenience of expression, the CLD material layouts divided into two equal subareas $S_{1}^{ke}$ and $S_{2}^{ke}$ based on the distributions of elemental kinetic energy are designated as Case 1, and the CLD material layouts divided into two equal subareas $S_{1}^{se}$ and $S_{2}^{se}$ based on the distributions of elemental strain energy are named Case 2. A thickness variables vector $h=\left\{\begin{array}{cccc} h_{v1} & h_{c1} & h_{v2} & h_{c2} \end{array}\right\}$ corresponding to the subareas $S_{1}^{ke}$($S_{1}^{se}$) and $S_{2}^{ke}$($S_{2}^{se}$) can be defined. Then, optimal thickness reconfigurations of VEM and CLM located at the subareas are searched for using the genetic algorithm (GA). In the evolutionary process, the additional mass fractions are constrained from the initial values $\delta_{i}$ = 60.42%, 49.48%, and 39.02%, to the target value $\delta_{2}$ = 50%, respectively. For the layout shown in Figure 5d, it is evident that the optimal thickness is 0.2 mm for all the elements, while the additional mass fraction is changed from $\delta_{1}$ = 25% to $\delta_{2}$ = 50%.

Table 2 and Table 3 illustrate the optimal thickness reconfigurations of VEM and CLM, as well as the corresponding sound power and MDRs for the two cases. Comparing Table 2 and Table 3 with Table 1, it is very clear that a smaller sound power and larger MDRs can be obtained while the thicknesses of VEM and CLM are optimally reconfigured. The minimum sound powers (104.84 dB and 107.06 dB) are obtained, coinciding with the maximum MDRs (0.118 and 0.099), for Case 1 and Case 2. The optimized thickness reconfigurations of CLD materials with the minimum sound power of the two cases have different initial mass fractions of 49.48% and 25.00%, which are different from the optimal mass fraction 60.42% obtained in first-level optimization. Hence, it can be concluded that the optimal position layouts of CLD materials with the minimum sound power are not always the optimal topology configuration for CLD structures, while size optimization is further considered. It is also noted from Table 3 that the optimized thickness reconfigurations of CLD materials with a smaller sound power do not always induce larger MDRs.

Furthermore, comparing the sound power in Table 2 and Table 3, it can be seen that a larger sound power reduction can be further achieved while the initial optimal position layouts of CLD materials are divided based on the distributions of average normalized elemental kinetic energy. The results (bold values of *h_v_*_1_ and *h_c_*_1_ in Table 2) suggest that more CLD materials should be pasted on the subareas with a higher elemental kinetic energy, and less CLD materials should be pasted on the areas with a lower elemental kinetic energy. However, a similar conclusion cannot be inferred from Table 3.

#### 5.2.2. Thickness Optimization for Case 3 and Case 4

**Figure 9:**
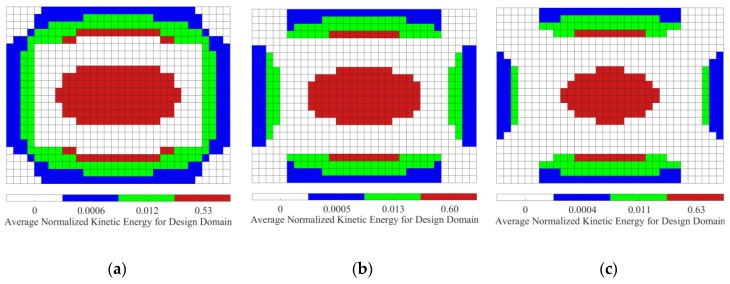
Diagram of the three subareas divided based on distributions of the average normalized element kinetic energy (‘red’, ‘green’, and ‘blue’ colors denote subareas $S_{1}^{ke}$, $S_{2}^{ke}$, and $S_{3}^{ke}$, respectively, with a descending order of average elemental kinetic energy). (**a**) AMF = 60.42%; (**b**) AMF = 49.48%; (**c**) AMF = 39.02%.

**Figure 10:**
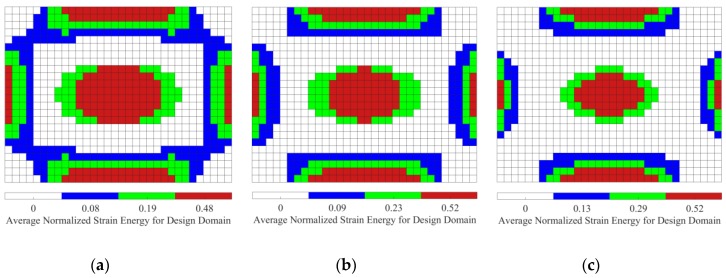
Diagram of the three subareas divided based on distributions of the average normalized element strain energy (‘red’, ‘green’, and ‘blue’ colors denote subareas $S_{1}^{se}$, $S_{2}^{se}$, and $S_{3}^{se}$, respectively, with a descending order of average elemental strain energy). (**a**) AMF = 60.42%; (**b**) AMF = 49.48%; (**c**) AMF = 39.02%.

In fact, for the optimized position layouts of CLD materials, it may be unreasonable to divide the layouts into too many subareas in practical applications due to the computational effort required for searching for optimal thickness reconfigurations or the manufacturing effort. Here, for the purpose of comparison, the initial design domains shown in Figure 5a–c are further divided into three equal subareas based on the same rules presented in Section 5.2.1, as illustrated in Figure 9 and Figure 10. These subareas are indicated by $S_{1}^{ke}$($S_{1}^{se}$), $S_{2}^{ke}$($S_{2}^{se}$), and $S_{3}^{ke}$($S_{3}^{se}$), denoted using a ‘red’ color, ‘green’ color, and ‘blue’ color, respectively. Similarly, the corresponding CLD material layouts are designated as Case 3 and Case 4 for simplicity of expression. A thickness variable vector $h=\left\{\begin{array}{cccccc} h_{v1} & h_{c1} & h_{v2} & h_{c2} & h_{v3} & h_{c3} \end{array}\right\}$ for the subareas $S_{1}^{ke}$($S_{1}^{se}$), $S_{2}^{ke}$($S_{2}^{se}$), and $S_{3}^{ke}$($S_{3}^{se}$) is also defined. The procedures employed for searching for optimal thickness reconfigurations of VEM and CLM for these subareas are carried out, in which the same constraint settings used in Section 5.2.1 are employed.

Table 4 and Table 5 list the optimal thickness reconfigurations of VEM and CLM for the three equal subareas, the corresponding sound power, and the MDRs for Case 3 and Case 4. It is also apparent that a smaller sound power and larger MDRs can be obtained for the two cases. Comparing the results of Case 3 and Case 1, the minimum sound power (105.44 dB) for Case 3 is a little greater than that (104.84 dB) for Case 1, and the corresponding position layouts of CLD materials are not the same. This implies that a smaller sound power cannot be definitely obtained, while the thicknesses of CLD materials are optimally reconfigured with considerations of optimized position layouts of CLD materials being divided into more subareas. Furthermore, as demonstrated in Table 4, more CLD materials should be pasted on the subareas with an average largest kinetic energy and less CLD materials should be pasted on the areas with a smaller average elemental kinetic energy with a given additional mass fraction. Besides, an interesting result can be found, in that the optimal thickness of VEM is slightly larger than or equal to the optimal thickness of CLM in the subareas of a higher elemental kinetic energy (*h_v_*_1_ and *h_c_*_1_ with a single underline in Table 2 and Table 4), and that (*h_v_*_1_ and *h_c_*_1_ with a double underline in Table 2 and Table 4) is opposite in the subareas of a smaller elemental kinetic energy. Comparing the results of Case 4 and Case 2, the sound power values for Case 4 are all smaller than those for Case 2, and the position layouts of CLD materials inducing a minimum sound power are also not the same. In addition, the optimal thickness reconfigurations seem to have nothing to do with the distributions of elemental strain energy. Besides, comparing the tendencies of sound power and MDRs in Table 4 and Table 5, it is clear that the optimization procedure minimizing sound power does not ensure a minimum vibration level of the vibrating structure.

As a summation from the four cases, some general conclusions can be obtained: (1) While the thicknesses of VEM and CLM are optimally reconfigured by taking optimized position layouts of CLD materials as the initial design, a smaller sound power and vibration level can be further achieved; (2) the optimized position layouts of CLD materials, which are divided into subareas based on the distribution of elemental kinetic energy, can be considered as a better initial design for further reconfiguring the thickness of CLD materials; and (3) for CLD structures, the optimization procedure minimizing the sound power does not ensure a minimum vibration level.

#### 5.2.3. Further Analysis of Sound Power for Case 1 and Case 2

Taking Case 1 and Case 2 in Section 5.2.1 as examples, the sound radiation power is further analyzed while only one of the thicknesses of VEM and CLM located in the two subareas *S*_1_ (i.e., $S_{1}^{ke}$, $S_{1}^{se}$) and *S*_2_ (i.e.,$S_{2}^{ke}$, $S_{2}^{se}$) are reconfigured and the other is locked to be the initial value (0.1 mm). Firstly, the target additional mass fractions are controlled to be initial mass fractions $\delta_{2}=\delta_{i}$ = 60.42%, 49.48%, and 39.02%, as in Figure 5a–c, in the optimization procedure. Hence, the thickness of VEM or CLM located in subareas *S*_1_ and *S*_2_ can be decided by the following formulation:

(24) $$n_{1}h_{1}^{v,c}+n_{2}h_{2}^{v,c}=nh^{v,c},\;n_{1}+n_{2}=n$$

in which, *n*_1_ and *n*_2_ denote the element number of subareas *S*_1_ and *S*_2_, respectively; *n* is the total element number decided by the target additional mass fraction; *h*_1_ and *h*_2_ are the thickness of VEM or CLM of subareas *S*_1_ and *S*_2_, respectively; and *h* is the corresponding initial thickness.

**Figure 11:**
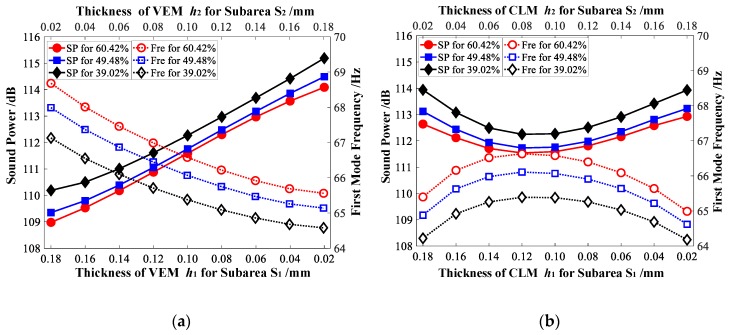
Relationship between sound power/natural frequencies of the CLD/plate and the thickness of viscoelastic materials (VEM) and constrained layer materials (CLM) for Case 1. (**a**) Sound power/natural frequencies vs. *h_v_*; (**b**) Sound power/natural frequencies vs. *h_c_*.

For Case 1, Figure 11a illustrates the sound power and natural frequencies of vibrating CLD/plate, while the thickness of VEM within subareas $S_{1}^{ke}$ and $S_{2}^{ke}$ varies in the opposite way and the thickness of CLM are set to be the initial value. It can be concluded that a greater thickness of VEM for subarea $S_{1}^{ke}$ with a higher element kinetic energy (smaller thickness of VEM for subarea $S_{2}^{ke}$ with a smaller element kinetic energy), smaller sound power, and larger natural frequencies can be achieved. Meanwhile, Figure 11b shows the relationship between the sound power/natural frequencies of CLD/plate and the thickness of CLM within subareas $S_{1}^{ke}$ and $S_{2}^{ke}$. It can be seen that the sound power decreases first and then increases as the thickness of CLM in subarea $S_{1}^{ke}$ decreases, but the variation of natural frequencies exhibits the opposite trend. In addition, as demonstrated in Figure 11, larger sound power reduction and natural frequencies can always be achieved with a larger added mass of CLD materials.

**Figure 12:**
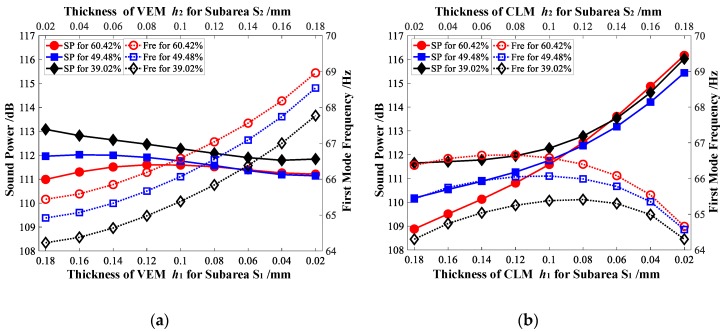
Relationship between sound power/natural frequencies of the CLD/plate and the thickness of VEM and CLM for Case 2. (**a**) Sound power/natural frequencies vs. *h_v_*; (**b**) Sound power/natural frequencies vs. *h_c_*.

For Case 2, Figure 12a shows the sound power and natural frequencies of vibrating CLD/plate, while the thickness of VEM within subareas $S_{1}^{se}$ and $S_{2}^{se}$ varies in the opposite way and the thicknesses of CLM are set to be the initial value. As shown as Figure 12a, the case is different from Figure 11a in that the sound power changes are small and display a slow downward trend, but the natural frequencies of CLD/plate increase while the thickness of VEM in the subarea $S_{1}^{se}$ decreases. Meanwhile, the relationships between the sound power and natural frequencies of CLD/plate and the thickness of CLM arranged in subareas $S_{1}^{se}$ and $S_{2}^{se}$ are given in Figure 12b. Compared with Figure 11b, it can be seen that there is a difference in that the sound power increases as the thickness of CLM in the subarea $S_{1}^{se}$ decreases, but natural frequencies still display a tendency to first slowly increase and then decrease. In addition, we can see that from Figure 12, with a larger added mass of CLD materials (larger additional mass fraction), larger natural frequencies can still be achieved, but larger sound power reduction cannot always be obtained, with the thickness *h_v_* and *h_c_* in subarea $S_{1}^{se}$ decreasing (*h_v_* and *h_c_* in subarea $S_{2}^{se}$ increasing simultaneously).

**Figure 13:**
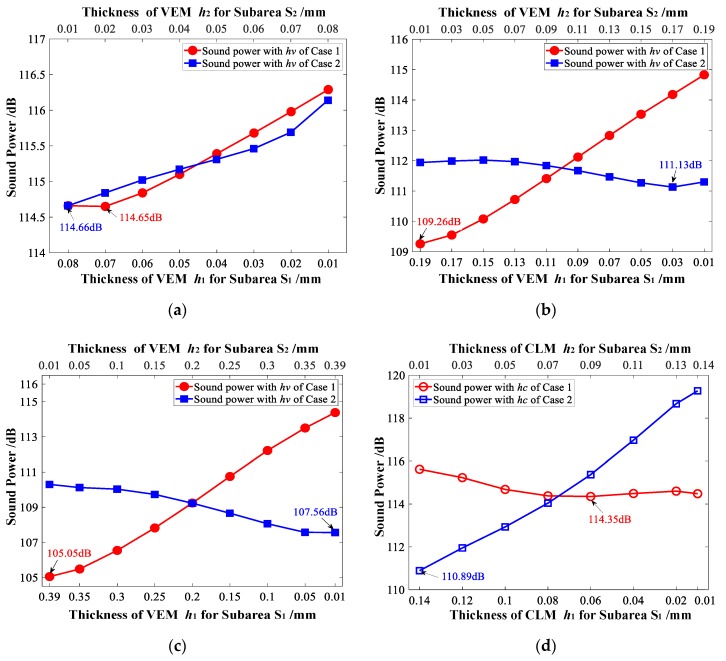

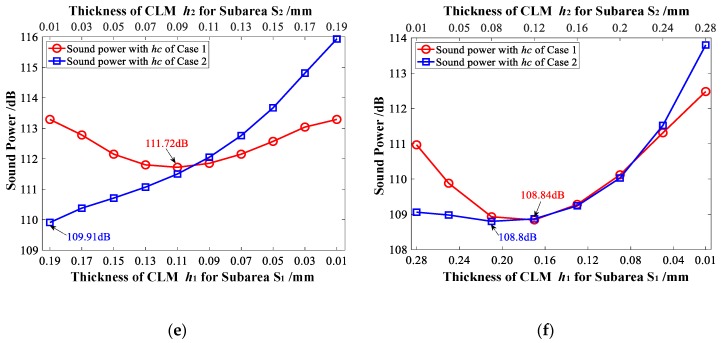
Relationship between sound power and the thickness of VEM and CLM for Case 1 and Case 2 with the target additional mass fraction $\delta_{2}$ = 50%. (**a**) Sound power vs. *h_v_* while $\delta_{i}$ = 60.42%; (**b**) sound power vs. *h_v_* while $\delta_{i}$ = 49.48%; (**c**) sound power vs. *h_v_* while $\delta_{i}$ = 39.02%; (**d**) sound power vs. *h_c_* while $\delta_{i}$ = 60.42%; (**e**) sound power vs. *h_c_* while $\delta_{i}$ = 49.48%; (**f**) sound power vs. *h_c_* while $\delta_{i}$ = 39.02%.

Furthermore, for Case 1 and Case 2, the sound power of the vibrating CLD/plate is analyzed, while the target additional mass fraction $\delta_{2}$ = 50% is achieved by varying the thickness of VEM or CLM of *S*_1_ and *S*_2_ separately. The thicknesses of VEM or CLM of *S*_1_ and *S*_2_ are also decided using Equation (24), and the minimum value is set to be 0.01 mm. The relationship between the sound power of vibrating CLD/plate and the thicknesses of VEM arranged in subareas *S*_1_ and *S*_2_ is illustrated in Figure 13a–c. It shows that the sound power displays a decreasing trend while the thickness of VEM located in higher elemental kinetic energy subarea *S*_1_ increases for Case 1, but similar rules cannot be obtained for Case 2. It can also be found that while more VEM is arranged in the higher elemental kinetic energy subarea *S*_1_, a smaller sound power for Case 1 (114.65 dB < 114.66 dB, 109.26 dB < 111.13 dB, and 105.05 dB < 107.56 dB, shown in Figure 13a–c, respectively) can always be achieved. Some similar conclusions can be found and inferred from Figure 13d–f. The sound power also displays a decreasing trend while the thickness of CLM located in higher elemental strain energy subarea *S*_1_ increases for Case 2, but similar rules cannot be obtained for Case 1. A smaller sound power for Case 2 (110.89 dB < 114.35 dB, 109.91 dB < 111.72 dB, and 108.8 dB < 108.84 dB, shown in Figure 13d–f, respectively) can always be achieved while more CLM is arranged in the higher elemental strain energy subarea *S*_1_.

Besides, the sound power illustrated in Figure 13, which is obtained by taking the same position layout of CLD materials as the initial design, is further analyzed. Hence, comparing the sound power in Figure 13a,d, it can be seen that a smaller sound power (110.89 dB) is achieved while more CLM material is pasted in the higher elemental strain energy subarea *S*_1_ for Case 2. However, comparing the sound power listed in Figure 13b,e and Figure 13c,f, a smaller sound power (109.26 dB and 105.05 dB) can be achieved by arranging more VEM materials in the higher elemental kinetic energy subarea *S*_1_. The minimum sound power of 105.05 dB is obtained from Case 1, in which only the thickness of VEM is reconfigured for the position layout shown in Figure 5c.The analysis implies that even if only the thickness of VEM or CLM is required to optimally reconfigure, the optimized position layouts of CLD materials, which are divided into subareas based on the distribution of elemental kinetic energy, should still be considered as a better initial design to suppress the sound power.

## 6. Conclusions

This paper presents a systematic methodology for designing position layouts and thickness configurations of CLD materials for suppressing the sound power of vibrating structures. Considering different additional mass fractions as constraints, a two-level optimization model for CLD/plate is formulated. In first-level optimization, a modified bi-directional evolutionary structural optimization (BESO) method, which is characterized by a relaxed element removal/addition criterion, is employed to obtain several optimal position layouts of CLD materials pasted on the base structure. The sound power sensitivity based on sound radiation modes is formulated to conduct the evolution directions of CLD materials. In second-level optimization, the optimized position layouts of CLD materials are taken as the initial design, and two strategies which are based on distributions of the averaged normalized elemental kinetic energy and strain energy are proposed to divide the layout into several subareas. Then, the thicknesses of viscoelastic materials (VEM) and constrained layer materials (CLM) of the subareas are reconfigured optimally using the genetic algorithm (GA), while the additional mass fractions are changed from relaxed values to target values.

Numerical examples firstly demonstrate that the modified BESO is competent for the position optimization of CLD materials and causing the computational effort to decrease. Both the sound power and vibration level can be effectively suppressed with optimized partially-covered CLD layouts and an optimal additional mass fraction leading to minimum sound power can be found. Then, the two proposed strategies are used to divide optimal position layouts into two or three subareas, and the thicknesses of CLD materials treated on the subareas are optimally reconfigured using GA. The numerical results show that both the sound power and vibration level are further suppressed, but the optimization procedure minimizing sound power does not ensure a minimum vibration level. An important issue is also demonstrated, in that the optimized position layouts of CLD materials, which are divided into subareas based on the distribution of elemental kinetic energy, can be considered as a better initial design for further size optimization. The results also suggest that optimal position layouts of CLD materials obtained in first-level optimization are not always the optimal topology configurations of CLD structures for size optimization, and more CLD materials should be pasted on the subareas with a higher elemental kinetic energy. Finally, the independent influences of the thickness of VEM or CLM located in the subareas on the sound power are further analyzed. As a supplementary conclusion, the results illustrate that more VEM should be arranged in the subarea with a higher average elemental kinetic energy, and more CLM seems to be treated on the subarea with a higher average elemental strain energy, while the two thicknesses are reconfigured separately.

## Figures and Tables

**Table 1:** Sound power and modal damping ratios (MDRs) for optimal CLD layouts and the fully treated CLD/plate.

Mass Fraction of CLD Materials	Sound Power/dB	MDRs
100.00%	113.27	0.048
60.42%	111.59	0.060
49.48%	111.76	0.058
39.02%	112.27	0.056
25.00%	113.70	0.049

**Table 2:** Optimal thickness reconfigurations, sound power, and MDRs for Case 1 in second-level optimization.

Initial Mass Fraction	Actual Mass Fraction	$\mathrm{Subarea}\;S_{1}^{ke}$		$\mathrm{Subarea}\;S_{2}^{ke}$		Sound Power/dB	MDRs
		*h_v_*_1_/mm	*h_c_*_1_/mm	*h_v_*_2_/mm	*h_c_*_2_/mm		
60.42%	49.96%	**0.19**	**0.14**	$\underline{\underline{0.01}}$	$\underline{\underline{0.01}}$	106.29	0.103
49.48%	49.95%	**0.19**	**0.19**	$\underline{\underline{0.01}}$	$\underline{\underline{0.01}}$	**104.84**	**0.118**
39.02%	49.50%	**0.2**	**0.2**	$\underline{\underline{0.01}}$	$\underline{\underline{0.08}}$	105.70	0.109
25.00%	50.00%	0.2	0.2	$\underline{\underline{0.2}}$	$\underline{\underline{0.2}}$	107.06	0.099

Note 1: Case 1—Optimal CLD material layouts divided into two subareas based on the distribution of elemental kinetic energy.

**Table 3:** Optimal thickness reconfigurations, sound power, and MDRs for Case 2 in second-level optimization.

Initial Mass Fraction	Actual Mass Fraction	$\mathrm{Subarea}\;S_{1}^{se}$		$\mathrm{Subarea}\;S_{2}^{se}$		Sound Power/dB	MDRs
		*h_v_*_1_/mm	*h_c_*_1_/mm	*h_v_*_2_/mm	*h_c_*_2_/mm		
60.42%	49.96%	0.19	0.14	0.01	0.01	108.14	0.084
49.48%	49.95%	0.19	0.19	0.01	0.01	107.74	0.085
39.02%	49.98%	0.07	0.15	0.2	0.11	108.09	0.089
25.00%	50.00%	0.2	0.2	0.2	0.2	**107.06**	**0.099**

Note: Case 2—Optimal CLD material layouts divided into two subareas based on the distribution of elemental strain energy.

**Table 4:** Optimal thickness reconfigurations, sound power, and MDRs for Case 3 in second-level optimization.

Initial Mass Fraction	Actual Mass Fraction	$\mathrm{Subarea}\;S_{1}^{ke}$		$\mathrm{Subarea}\;S_{2}^{ke}$		$\mathrm{Subarea}\;S_{3}^{ke}$		Sound Power/dB	MDRs
		*h_v_*_1_/mm	*h_c_*_1_/mm	*h_v_*_2_/mm	*h_c_*_2_/mm	*h_v_*_2_/mm	*h_c_*_2_/mm		
60.42%	49.96%	**0.2**	**0.2**	0.01	0.01	$\underline{\underline{0.03}}$	$\underline{\underline{0.04}}$	**105.44**	0.108
49.48%	49.90%	**0.2**	**0.2**	0.01	0.01	$\underline{\underline{0.03}}$	$\underline{\underline{0.12}}$	105.63	0.107
39.02%	49.73%	**0.2**	**0.18**	0.2	0.18	$\underline{\underline{0.01}}$	$\underline{\underline{0.01}}$	105.68	**0.111**
25.00%	50.00%	0.2	0.2	0.2	0.2	$\underline{\underline{0.2}}$	$\underline{\underline{0.2}}$	107.06	0.099

Note: Case 3—Optimal CLD material layouts divided into three subareas based on the distribution of elemental kinetic energy.

**Table 5:** Optimal thickness reconfigurations, sound power, and MDRs for Case 4 in second-level optimization.

Initial Mass Fraction	Actual Mass Fraction	$\mathrm{Subarea}\;S_{1}^{se}$		$\mathrm{Subarea}\;S_{2}^{se}$		$\mathrm{Subarea}\;S_{3}^{se}$		Sound Power/dB	MDRs
		*h_v_*_1_/mm	*h_c_*_1_/mm	*h_v_*_2_/mm	*h_c_*_2_/mm	*h_v_*_2_/mm	*h_c_*_2_/mm		
60.42%	49.65%	**0.01**	**0.01**	**0.2**	**0.2**	**0.08**	**0.01**	107.42	0.105
49.48%	49.86%	**0.04**	**0.12**	**0.01**	**0.01**	**0.2**	**0.2**	106.77	0.108
**39.02%**	49.75%	**0.01**	**0.01**	**0.2**	**0.2**	**0.2**	**0.17**	**106.02**	**0.120**
25.00%	50.00%	0.2	0.2	0.2	0.2	0.2	0.2	107.06	0.099

Note: Case 4—Optimal CLD material layouts divided into three subareas based on the distribution of elemental strain energy.
